# Clinical study on the characteristics and related influencing factors of deliberate drug ingestion in adolescents

**DOI:** 10.12669/pjms.40.8.8828

**Published:** 2024-09

**Authors:** Jiangtao Ma, Yanli Zhao, Jing Li, Ying Zhang, Jie Chen

**Affiliations:** 1 Jiangtao Ma, Department of Urology Surgery, Baoding Hospital of Beijing Children’s Hospital Affiliated to Capital Medical University, Baoding 071000, Hebei, China; 2 Yanli Zhao, Department of Urology Surgery, Baoding Hospital of Beijing Children’s Hospital Affiliated to Capital Medical University, Baoding 071000, Hebei, China; 3 Jing Li, Department of Urology Surgery, Baoding Hospital of Beijing Children’s Hospital Affiliated to Capital Medical University, Baoding 071000, Hebei, China; 4 Ying Zhang, Department of Urology Surgery, Baoding Hospital of Beijing Children’s Hospital Affiliated to Capital Medical University, Baoding 071000, Hebei, China; 5 Jie Chen, Department of Urology Surgery, Baoding Hospital of Beijing Children’s Hospital Affiliated to Capital Medical University, Baoding 071000, Hebei, China

**Keywords:** Adolescence, Children, Deliberate Drug Ingestion, Clinical Characteristics, Influencing Factors

## Abstract

**Objective:**

To analyze the characteristics of deliberate drug ingestion in adolescents and its related influencing factors.

**Method:**

This was a retrospective study. A total of 158 cases of deliberate drug ingestion as observation group and 160 cases of other diseases in adolescents as control group were treated in the Emergency Department of Baoding Hospital of Beijing Children’s Hospital Affiliated to Capital Medical University from January 2020 to December 2022. The clinical characteristics of adolescents who engaged in deliberate drug ingestion were analyzed, and various factors that could potentially influence deliberate drug ingestion in adolescents were subjected to both univariate and multivariate analysis.

**Result:**

There was a progressive increase in the number of patients presenting with mental health issues year by year. Univariate analysis showed that family type, guardian’s education level, place of residence, whether they were only children, parents’ knowledge of medication, awareness of medication safety, depression/anxiety, negative life events, and social support were risk factors for deliberate drug ingestion in adolescents (all p<0.05). Logistic regression analysis showed that family type, parents’ knowledge of medication, awareness of medication safety, whether they were depressed/anxious, negative life events, and social support were independent risk factors for deliberate drug ingestion in adolescents (p<0.05).

**Conclusion:**

The incidence of deliberate drug ingestion in adolescents is increasing year by year, and their behavior is influenced by multiple factors. Interventions should be targeted at controllable influencing factors to prevent or reduce deliberate drug ingestion in adolescents.

## INTRODUCTION

Adolescence is a crucial period characterized by rapid physical and psychological development in the human body. During this stage, children undergo significant changes in both their mental and physical aspects. They may become more sensitive, experience emotional instability, and have reduced communication with their parents. These changes can bring about various confusions and challenges for adolescents, increasing their vulnerability to conditions such as anxiety, depression, self-harm, and even suicide.^1^-^3^ Data reveals that approximately 24.6% of adolescents are affected by depression.^4^ Since adolescents are still in the process of cognitive development, their ability to cope with anxiety or depression may be limited. Consequently, these conditions can significantly impact their learning abilities, daily functioning, psychosocial development, and interpersonal relationships. Moreover, the effects can extend to negatively affect their families as well.

During puberty, some adolescents may resort to self-harm or suicide attempts as a result of anxiety or depression. Research indicates that the rate of self-harm behavior among Chinese adolescents is approximately 22.37%, and suicide has become the third leading cause of death in the age group of 10-25.^5^ Studies have further highlighted that the peak period for adolescent suicide falls between the ages of 10 and 19, with around 67% of adolescents utilizing drug overdose as a method.^6^ Adolescence is a period of approaching physical and psychological maturity, characterized by a complex inner world and an unstable worldview. External factors can easily influence them. Moreover, this stage is marked by physiological and psychological upheavals, rendering them more susceptible to extreme emotions and behaviors, including suicide, in response to stressors.

However, there is currently a significant lack of attention focused on adolescent suicide, particularly in terms of conducting studies that delve into the inner experiences of adolescents who deliberately ingest drugs as a means of suicide. This study aimed to comprehensively analyze the clinical data of adolescents who deliberately ingested drugs in our hospital. The objective was to identify and summarize the characteristics and relevant influencing factors associated with deliberate drug ingestion during adolescence. These findings will serve as a foundation for the development of prevention and intervention measures tailored to this specific population.

## METHODS

This was a retrospective study. One hundred and fifty-eight adolescent patients who intentionally ingested drugs and were admitted to the emergency department of Baoding Hospital of Beijing Children’s Hospital Affiliated to Capital Medical University between January 2020 and December 2022 were selected as the observation group for this study. Additionally, 160 other adolescent patients were selected as the control group. All the subjects were retrieved from electronic medical record systems undergoing Baoding Hospital of Beijing Children’s Hospital Affiliated to Capital Medical University.

### Ethical Approval

The study was approved by the Institutional Ethics Committee of Baoding Hospital of Beijing Children’s Hospital Affiliated to Capital Medical University (No.: 2023020; date: March 02, 2023), and written informed consent was obtained from all guardians of the participants.

### Inclusion criteria:

- Clear history of drug ingestion
- Laboratory findings indicating drug presence or specific enzymatic abnormalities, such as abnormal gastric contents or routine blood, urine, or stool tests.
- Stable emotional and medical condition, clear consciousness, and no cognitive or language communication barriers during interviews.
- Age between 0-18 years.

### Exclusion criteria:

- Patients with serious physical diseases;
- Patients with other mental diseases;
- Patients with intellectual disability or severely impaired cognitive function could not complete the interview.

The observation group consisted of individuals aged 10-17 years, with an average age of 12.87±1.32 years, including 35 males and 123 females. The control group consisted of individuals aged 11-16 years, with an average age of 12.95±1.23 years, including 42 males and 118 females. There were no significant differences in age and gender between the two groups (p>0.05). Table-I

**Table-I:** Comparison of General Characteristics between Two Groups.

Variables	Observation (n=158)	Control (n=160)	t/c^2^	P
Age (years)	12.87±1.32	12.95±1.23	-0.580	0.563
Gender (Male/Female, n)	35/123	42/118	0.728	0.394

### Research Methods

Based on the literature on factors related to substance abuse, a survey questionnaire was been designed. The questionnaire included the following details: name, gender, age, grade, family type, parental education level, place of residence, only child status, parental knowledge of medication, and awareness of medication safety. The Center for Epidemiologic Studies Depression Scale (CES-D) from the Epidemiology Center was utilized to assess the level of depression in the affected adolescent patients. The evaluation criteria encompass depressive mood, interpersonal relationships, physical symptoms, and positive emotions.

The scale consists of 20 assessment items, each with four rating levels, resulting in a total score of 60. Scores ranging from 0-16 indicate no depression, 17-22 indicate possible depression, 23-27 indicate likely depression, and 28-60 indicate severe depression. The Multidimensional Anxiety Scale for Children (MASC) was employed to evaluate the anxiety symptoms experienced by individuals over the past week. The scale utilizes a 0-3 rating scale and comprises four subscales: physical symptoms, social anxiety, separation anxiety, and harm avoidance. The cumulative score provides a total score, with higher scores indicating more severe anxiety symptoms. A score greater than 60 indicates overall significant anxiety symptoms, while a score greater than 75 indicates extremely severe anxiety symptoms. All the questionnaire was given to the participants or their guardians for investigation, and then collected in about five minutes.

The Adolescent Self-Rating Life Events Checklist (ASLEC) was used to assess the frequency and intensity of life events and stress experienced by adolescents in the past year. It is a commonly used self-assessment scale for evaluating stressors in adolescent life. It consists of 27 items, including six dimensions: interpersonal relationships, academic pressure, punishment, loss, health and adaptation, and others. The individual rates the impact of each event on a five point scale, with higher scores indicating more severe negative life events experienced by the patient. The Adolescent Social Support Scale (ASSS) consists of 18 items and assesses three dimensions: subjective support, objective support, and support utilization. It uses a five point rating scale, where higher scores indicate greater social support received and positive attention from others. It is primarily used to evaluate social support and positive attention from others among adolescents.

### Quality Control

Trained researchers carried out the questionnaire survey, providing clear explanations of the content and filling instructions to the participants. After obtaining informed consent, the participants independently completed the questionnaires, and the researchers collected the completed questionnaires on-site. Data entry was conducted based on the participants’ actual responses, and a second entry check was performed to identify and rectify any inconsistencies in the data. In cases where inconsistent data were found, the original questionnaires were referred to using the assigned numbers, and the data were re-entered accordingly. Questionnaires with a missing rate exceeding 5% or with obviously consistent responses were excluded.

### Statistical Analysis

Data processing and statistical analysis were performed using SPSS 22.0 software. Count data were presented as percentages (%), and c² test was used for comparisons. Measurement data were expressed as `x±s, and the independent sample *t* test was used for comparisons. The confidence interval was 95%. Logistic regression analysis was used for multifactor analysis, with a significance level of p<0.05 indicating statistical significance.

## RESULTS

Characteristics of Deliberate Drug Ingestion. Over a period from 2020 to 2022, a total of 158 cases of deliberate medication among adolescent patients were treated, and the number of cases exhibited an increasing trend each year (34, 54, 70). Among these cases, there were more girls than boys, with a ratio of 123:35. There was a progressive increase in the number of patients with mental health issues such as depression, anxiety, and rebellion, with the respective figures for each year being 13, 20, and 28 cases. The primary types of medications taken deliberately by these patients included antipsychotic drugs, with 27 cases involving quetiapine, 15 cases involving alprazolam, 14 cases involving cold medicine, 11 cases involving lorazepam, 11 cases involving pesticides, three cases involving rodenticides, three cases involving clonidine, and three cases involving cephalosporin antibiotics.

The clinical manifestations of the patients after drug ingestion mainly included gastrointestinal symptoms such as abdominal pain, nausea, and vomiting, and some patients also showed dizziness, drowsiness, and fatigue. Except for one patient who ingested paraquat and was subsequently transferred to Beijing Children’s Hospital, all other patients showed improvement and were discharged after receiving gastric lavage and supportive care. Fortunately, no fatalities were reported among the cases. Univariate analysis was performed to examine factors associated with deliberate drug ingestion in adolescents. The findings indicated that there was no statistically significant difference between the two groups in terms of the factor “whether family members are medical personnel”(p>0.05). However, significant differences were observed between the two groups regarding family type, educational level of guardians, place of residence, only child status, parental knowledge of medication, awareness of medication safety, depression/anxiety, negative life events, and social support (all p<0.05). Table-II.

**Table-II:** Univariate Analysis of Factors Related to Deliberate Drug Ingestion in Adolescents.

Variables		Groups		c^2^	P

		Observation	Control		
Family member being a healthcare professional	Yes	42	51	1.076	0.300
	No	116	109		
Family type	Single parent	71	42	12.118	0.000
	Both parents	87	118		
Parental education level	Low	70	51	5.210	0.022
	High	88	109		
Place of residence	Rural	75	58	4.112	0.043
	Urban	83	102		
Only child	Yes	95	75	5.611	0.018
	No	63	85		
Parental knowledge of medication	Weak	102	82	5.774	0.016
	Strong	56	78		
Awareness of medication safety	Weak	108	91	4.473	0.034
	Strong	50	69		
Depression/Anxiety	Yes	61	35	10.561	0.001
	No	97	125		
Negative life events	Severe	58	38	6.335	0.012
	Not severe	100	122		
Social support	Weak	80	52	10.766	0.001
	Strong	78	108		

The logistic regression analysis results revealed that family type, parental knowledge of medication, awareness of medication safety, depression/anxiety, negative life events, and social support were identified as independent risk factors influencing deliberate drug ingestion in adolescent children (p<0.05). However, the educational level of guardians, place of residence, and only child status was not found to be independent risk factors for deliberate drug ingestion in adolescent children (p>0.05). For more detailed information. Table-III.

**Table-III:** Logistic Regression Analysis of Factors Related to Deliberate Drug Ingestion in Adolescents.

Factors	Regression Coefficient	SE	Waldc^2^	P	OR	95%CI
Family type	1.681	0.530	10.067	0.002	5.373	1.902-15.182
Parental knowledge of medication	0.658	0.260	6.391	0.011	1.931	1.159-3.217
Awareness of medication safety	0.682	0.275	6.169	0.013	1.978	1.155-3.387
Depression/Anxiety	1.120	0.281	15.848	0.000	3.064	1.765-5.317
Negative life events	0.697	0.285	5.981	0.014	2.009	1.149-3.513
Social support	0.561	0.265	0.034	0.034	1.753	1.043-2.947

## DISCUSSION

The findings of this study reveal that the primary medication used in deliberate medication among adolescent children is the antidepressant sertraline, followed by sedatives and antipyretic drugs. In rural areas, the use of pesticides is relatively common, which can be attributed to higher pesticide usage rates, lower education levels, a lack of safety awareness and first aid knowledge, and reduced safety awareness among guardians.^7^

Research studies have indicated that deliberate medication in adolescent children is often triggered by factors such as academic pressure^8^-^10^, physiological challenges, complex social relationships, emotional disorders, and mental health issues. At first, deliberate medication in adolescent children did not receive sufficient attention. However, with the increasing number of cases and subsequent deaths related to this behavior, there has been a growing recognition of the significance of this issue.^11^

Adolescence is a crucial stage of human development characterized by significant physical and psychological changes, which can render adolescent children more vulnerable to psychological distress and emotional instability. Studies have shown notable individual differences in the characteristics of deliberate drug ingestion behavior among adolescents. In terms of gender distribution,^12^,^13^ females tend to engage in deliberate medication more frequently than males, aligning with the findings of this study and clinical reports.^14^ Domestic studies have indicated that deliberate drug ingestion primarily involves the use of antipyretic and analgesic drugs, antidepressants and psychotropic drugs, sedatives, and pesticides.

However, research on deliberate medication in adolescent children remains relatively limited, and international studies have shown overlapping medication types with commonly used deliberate drug ingestion.^15^,^16^ Deliberate drug ingestion is closely associated with individual and family environments. Most individuals engaging in deliberate drug ingestion have psychological health issues such as low self-esteem, self-importance, and emotional instability^17^, which can contribute to a sense of worthlessness and confusion. The family environment plays a significant role in influencing deliberate drug ingestion among adolescent children. Research suggests that negative family environments^18^, including family conflicts and parental divorce, negatively impact adolescents and increase their risk of deliberate drug ingestion. This study demonstrates that adolescent children from single-parent families are more prone to deliberate drug ingestion. In addition to psychological factors, individual characteristics, and family environment, the social environment also influences deliberate drug ingestion. Negative life events in daily life often led to negative emotions such as depression and anxiety.^19^,^20^ When adolescents face these intense negative emotions, deliberate drug ingestion becomes a primary coping mechanism.

The results of this study indicate that negative life events have an impact on deliberate drug ingestion behavior. Social support objectively helps adolescents access material and psychological assistance^21^, reducing the occurrence of self-harming behaviors. When social support is lacking, the likelihood of adolescents engaging in self-harm behaviors increases. Factors such as feelings of loneliness and strained interpersonal relationships may act as triggers for deliberate drug ingestion.^22^ The findings of this study demonstrate that social support acts as a protective factor against deliberate drug ingestion in adolescent children. Higher levels of social support correspond to a lower probability of deliberate drug ingestion, whereas lower levels of social support correspond to a higher probability.

The logistic regression analysis identified parental knowledge of medication and awareness of medication safety as influential factors in deliberate drug ingestion among adolescent children. Therefore, it is crucial to not only concentrate on the adolescents themselves but also disseminate knowledge regarding medication use and educate families about the significance of strict medication management. Moreover, providing education to both guardians and children about deliberate medication is essential in preventing its occurrence within the family.

Clinical research on deliberate drug ingestion in adolescent children should explore and study this issue from multiple perspectives. On one hand, attention should be given to the individual psychological health problems of adolescent children, providing timely psychological counseling and intervention measures to enhance their ability to cope with stress. On the other hand, the influence of family and social environments should also be considered, strengthening family education, school education, and social support to create a positive and healthy growth environment for adolescent children.

### Limitations

It includes a small number of observed cases and limited follow-up factors. Further research with larger sample sizes and additional follow-up factors is needed to further analyze the factors influencing deliberate drug ingestion in adolescent children.

## CONCLUSIONS

In conclusion, the incidence of deliberate drug ingestion in adolescents is increasing year by year, and their behavior is influenced by multiple factors. Deliberate drug ingestion among adolescent children is a complex psychological issue that requires interdisciplinary research and multi-level intervention measures. By conducting in-depth studies on its characteristics and related influencing factors, more effective prevention and intervention strategies can be provided to clinical doctors and mental health professionals, helping adolescent children overcome psychological difficulties, promote healthy development, and reduce the occurrence of deliberate drug ingestion.

### Authors’ Contributions:

**JM:** Carried out the studies, participated in collecting data, and drafted the manuscript, and are responsible and accountable for the accuracy or integrity of the work.

**YZ** and **JL:** Performed the statistical analysis and participated in its design.

**YZ** and **JC:** Performed the statistical analysis and participated in its design.

All authors have read and approved the final manuscript.
